# Arsenic Metabolism, Toxicity and Accumulation in the White Button Mushroom *Agaricus bisporus*

**DOI:** 10.3390/toxics10100554

**Published:** 2022-09-22

**Authors:** Owen Dong, Michael Powers, Zijuan Liu, Masafumi Yoshinaga

**Affiliations:** 1 Rochester Adams High School, Rochester, MI 48306, USA; 2 Department of Biological Sciences, Oakland University, Rochester, MI 48309, USA; 3 Department of Cellular Biology and Pharmacology, Herbert Wertheim College of Medicine, Florida International University, Miami, FL 33199, USA

**Keywords:** arsenic, toxicity, mushroom, metabolism, methylation, *Agaricus bisporus*, HPLC-ICP-MS, arsenite, arsenate, arsenobetaine

## Abstract

Mushrooms have unique properties in arsenic metabolism. In many commercial and wild-grown mushrooms, arsenobetaine (AsB), a non-toxic arsenical, was found as the dominant arsenic species. The AsB biosynthesis remains unknown, so we designed experiments to study conditions for AsB formation in the white button mushroom, *Agaricus bisporus*. The mushrooms were treated with various arsenic species including arsenite (As(III)), arsenate (As(V)), methylarsenate (MAs(V)), dimethylarsenate (DMAs(V)) and trimethylarsine oxide (TMAsO), and their accumulation and metabolism were determined using inductively coupled mass spectrometer (ICP-MS) and high-pressure liquid chromatography coupled with ICP-MS (HPLC-ICP-MS), respectively. Our results showed that mycelia have a higher accumulation for inorganic arsenicals while fruiting bodies showed higher accumulation for methylated arsenic species. Two major arsenic metabolites were produced in fruiting bodies: DMAs(V) and AsB. Among tested arsenicals, only MAs(V) was methylated to DMAs(V). Surprisingly, AsB was only detected as the major arsenic product when TMAsO was supplied. Additionally, AsB was only detected in the fruiting body, but not mycelium, suggesting that methylated products were transported to the fruiting body for arsenobetaine formation. Overall, our results support that methylation and AsB formation are two connected pathways where trimethylated arsenic is the optimal precursor for AsB formation.

## 1. Introduction

Ranked at the top of the United States Agency for Toxic Substances and Disease Registry (ATSDR)’s priority list of Hazardous Substances, arsenic is a Group 1 (proven) human carcinogen. Chronic exposure is associated with higher chances of a number of diseases [1,2,3]. Currently, the maximum contaminant level (MCL) for arsenic in drinking water set by the United States Environmental Protection Agency (EPA) is 10 ppb (parts per billion), a standard accepted by most other countries. Arsenic in drinking water remains to be a health threat. The elimination of arsenic from water and food is a significant challenge and new technology is urgently needed [4].

Arsenic is a pervasive metalloid and prevalent environmental toxin. Arsenic contamination of natural environments, including soil and ground water, is mainly a geographic process [5]. Natural geological occurring arsenicals are mostly inorganic, including trivalent arsenite (As(III)) and pentavalent arsenate (As(V)) [6]. In aquatic and soil environments, inorganic arsenicals can be metabolized into various arsenic products, majorly by microbes [7].

Many arsenic biotransformation pathways in microbes and mammals have been identified, including transport into/outside cells, the reduction/oxidation, methylation/demethylation, thiolation and many other modifications and degradations [8,9,10]. However, there are still many other arsenic biotransformation pathways not yet identified.

A common arsenic metabolism pathway found in microbes and humans is methylation [11]. Starting from inorganic arsenic, the arsenic S-Adenosyl methionine (SAM)-dependent methyltransferase (ArsM in microbes or AS3MT in animals) is capable of adding methyl-groups to arsenic to form mono-, di-, and tri-methylated arsenicals. The oxidative methylated arsenic species, including methylarsenate (MAs(V)), dimethylarsenate (DMAs(V)), and trimethylarsine oxide (TMAsO) are detected in many organisms and environments [11]. The reduced forms of these methylated species are in general unstable or in gas form, so they are not readily detected. The methylation process is, in general, deemed as a detoxification pathway as methylated products are typically less toxic, especially under aerobic environments.

Other unique arsenic metabolites have been reported, specifically arsenobetaine (AsB). AsB was first found as a major arsenic species in marine fish in the 1970s [12]. It is also a dominant arsenic species in excreta from humans with diets rich in fish, giving rise to the common term “fish arsenic” [13]. Unlike all other arsenic species, AsB is so far the only stable arsenical metabolite deemed as non-toxic and biologically safe. According to the Centers for Disease Control and Prevention (CDC), its estimated half-lethal dose (LD_50_) in mice is higher than 10 g per kilogram of body weight [14].

In addition to marine organisms, AsB was also found in multiple mushroom species [15,16,17,18,19,20]. Thus far, mushrooms seem to be the only terrestrial species known to form AsB from inorganic arsenic precursors. In many commercial and wild mushroom species, AsB is the most dominant arsenic species, some containing as much as 90% total arsenic in the form of AsB [19]. Mushrooms are therefore deemed as a unique organism in regard to arsenic metabolism through their ability to synthesize AsB.

Other types of common arsenic metabolites detected in mushrooms are methylated products, including mono-, di-, tri-, and even tetra-methylated species [17,18,21]. These species were discovered in both commercial mushroom products as well as cultured mushrooms under As(III) and As(V) treatment, confirming that methylation is one common metabolic pathway in mushrooms.

Most studies are species analysis in commercial or wild-grown mushrooms and a systematic investigation of arsenic species under various treatment is lacking, and a lab model for AsB formation is not well-established. In our study, we used a culturable white button mushroom *Agaricus bisporus* as a model organism to investigate various arsenic metabolism in order to identify conditions and mechanisms for AsB formation. It is also a species known to contain arsenic in the form of AsB [15].

Mushrooms have two development stages: the vegetative stage (mycelium or hyphae) and the reproductive stage (fruiting body). A previous study showed that in aseptically cultured *A. bisporus* mycelium, minimal arsenic metabolism occurred when As(V), MAs(V), DMAs(V), TMAsO, AsB or dimethylarsinoyl acetate was supplied [18]. Each of these arsenicals remained unchanged except for As(V), which was largely reduced into As(III). Unless AsB was supplied, no AsB was detected, suggesting that either the genes/enzymes for AsB formation were not expressed in the vegetative stage or the aseptic culture condition with organic liquid medium (Potato medium) did not satisfy the conditions for AsB formation.

In cultured *A. bisporus*, we designed experiments to explore conditions for AsB biosynthesis. Each of the five arsenic compounds, As(III), As(V), MAs(V), DMAs(V), and TMAsO was added to the compost containing mycelium, respectively. After fruiting, arsenic metabolites in the fruiting body were assayed. Our results showed that methylation is an active metabolism process and that the methylated species can be detected in the fruiting body. We also discovered that the most efficient precursor for AsB formation is the trimethylated arsenic: TMAsO. These results suggest that mushrooms use a unique pathway to synthesize AsB. We confirmed that AsB is only detected in the fruiting body, suggesting that its formation is a developmental stage-dependent process.

Various arsenic species detected in the environment have different levels of toxicity. The trivalent arsenic species are generally more toxic than their pentavalent counterparts. The inorganic arsenic species are toxic to both microbes and animals [14]. In this study, we also determined the toxicity of inorganic As(III) and As(V) as well as organic MAs(V) and DMAs(V) in the vegetative stage. We present data here to show that mycelium has different tolerance to various arsenic species with unique tolerances to inorganic arsenic species. We also compared total arsenic accumulation in the vegetative stage and reproductive stage and showed that the arsenic accumulation in these two stages share different patterns. These studies form a foundation for understanding how mushrooms respond to various environmental arsenicals and also propose new pathways for AsB biosynthesis.

## 2. Materials and Methods

### 2.1. Chemicals and Reagents

The chemicals and reagents used for the total arsenic and arsenic speciation analysis are sodium arsenite (As(III)), sodium arsenate (As(V)), methylarsonic acid (MAs(V)), dimethylarsinic acid (DMA(V)), trimethylarsine oxide (TMAsO), arsenocholine (AsC) and arsenobetaine (AsB). They were all acquired from Sigma-Aldrich (St. Louis, MO, USA) except TMAsO, which was purchased from Toronto Chemical Research (North York, ON, Canada). The standard for HPLC-ICP-MS speciation of these compounds was prepared accordingly.

### 2.2. Mycelium Culture under Aseptical Conditions

A white button mushroom fruiting kit was obtained from a commercial growing kit provider (Mushroom Mountain, Easley, SC, USA), from which the mycelium was purified. Mycelium was inoculated in liquid malt extract medium containing 1.5% malt extract (Sigma Aldrich, St. Louis, MO, USA) supplemented with 50 µg/L chloramphenicol, and cultured aseptically at 25 °C continuously for 7 days.

### 2.3. Mushroom Fruiting

The mushroom culture assembly kit contained pre-grown mycelium in the compost (culture matrix) and casing soil. Upon receiving, 300 g of culture matrix was aliquoted and mixed well with various arsenicals at the indicated final concentrations in a 15 × 15 cm cardboard box. A thin layer of activated casing soil was applied. Following the manufacturer’s guidelines, the assembly was misted twice a day and cultured at 20 °C. Fruiting usually starts in 2–3 weeks and the first flush is achieved in 3–4 weeks. The fruiting bodies (with diameter 3–5 cm) and mycelium-containing compost were collected, processed, and used for arsenic analysis.

### 2.4. Toxicity of Various Arsenicals in Vegetative Stage

To study the arsenic toxicity, arsenic compounds were added at the indicated final concentrations to a liquid medium (malt extract) containing inoculated mycelium for two weeks. The total growth of the mycelium was quantified. The mycelium was homogenized by an ultrasonic rod to a homogenized mycelium suspension which allows for OD_600_ to be measured in a microplate reader. The OD value was used as an indicator to compare the mycelium growth under different arsenic treatments. Similarly, the arsenic toxicity of *Saccharomyces cerevisiae* (budding Yeast BY4741, Horizon Discovery Biosciences Limited, Cambridge, Great Britain, UK) was also determined and used as a control to be compared with mushroom mycelium.

### 2.5. Total Arsenic Accumulation in Vegetative Stage and Fruiting Bodies

To study the arsenic accumulation in mycelium, mycelium (approximately 2 mg dry weight) was inoculated into a 4-mL liquid malt extract medium and cultured for 2 weeks. After mycelium growing up, As(III), As(V), MAs(V), DMA(V), or TMAsO was added to each culture at final concentrations of 100 μM each and incubated for additional 2 h. After incubation, each mycelium sample was filtered through cellulose membrane and washed three times in water. The membranes that contain mycelium were then digested with 0.3 mL of 70% nitric acid (≥99.999% trace metals basis) at 70 °C for 120 min, allowed to cool to room temperature and diluted with 6 mL of deionized water to produce a final concentration of nitric acid of 3.5%. Arsenic in the prepared samples was quantified by inductively-coupled plasma mass spectrometry (ICP-MS) (NexION 1000, Perkin Elmer, Waltham, MA, USA) [22]. ICP-MS analysis was operated with 1600 W radio-frequency (RF) power, 15 L/min plasma gas flow, 1.2 L/min auxiliary gas flow rate, 3 replicate measurements. The ICP-MS tuned using NexION Setup Solution (PerkinElmer) at the start of each analysis session to gain the optimal sensitivity. Arsenic solutions in the range of 1–50 ppb were prepared in 3.5% HNO_3_ using an arsenic standard (Ricca Chemical Company, Arlington, TX, USA). Total arsenic levels were calculated in the unit of μg As/mg dry weight.

To study arsenic accumulation in the fruiting body, mushrooms were allowed for fruiting under various arsenic treatments at final concentrations of 5 ppm in the culture matrix. The fruiting body was then collected and dried at 60 °C overnight. The dried mass was weighed, digested in 70% nitric acid, and total arsenic was quantified by ICP-MS as above described.

### 2.6. Extraction of Arsenic in Culture Matrix and Fruiting Body

Extraction of total arsenic for speciation studies used a previously published method [15]. The freshly collected fruiting body was homogenized in liquid nitrogen manually by molar and pestle. Then, 100 mg of the homogenized fruiting body was resuspended in 1 mL of 50% (*v*/*v*) aqueous methanol solution. The mixtures were vortexed and shaken end-over-end overnight at 4 °C. After centrifugation, the supernatant was filtered for arsenic speciation.

The arsenic in the compost was collected using a similar method. The culture matrix (5 g) was collected and suspended in 10 mL of 50% (*v*/*v*) aqueous methanol in a disposable polypropylene centrifuge tube. The mixtures were vortexed, shaken end-over-end overnight at 4 °C, and centrifuged. The supernatant was collected, filtered, and kept frozen until analysis.

### 2.7. Arsenic Speciation by HPLC-ICP-MS

Arsenic speciation was assayed by HPLC-ICP-MS according to previously reported procedures with slight modifications [15,23,24]. The HPLC system for arsenic speciation consisted of a degasser, an HPLC column oven, an HPLC binary pump and a standard autosampler (NexSar HPLC, Perkin Elmer). This system was connected to the intake of the ICP-MS (NexION 1000, PerkinElmer) for monitoring ^75^As (*m*/*z* = 75). A standard solution containing 1 µM of As(III), As(V), MAs(III), MAs(V), DMAs(V), TMAsO, AsC and AsB was freshly prepared and run at the beginning and end of each batch of sample analysis. Two commercial columns were adopted to analyze samples (injection volume: 100 µL): a cation exchange column (ChromSep IonoSpher 5 C, 100 × 3.0 mm (size), 5 µm (particle size), Agilent Technologies, Inc., Santa Clara, CA, USA) [20] with a mobile phase consisting of 20 mM pyridine (pH 2.7 adjusted by formic acid), and a C18 reverse-phase column (BioBasic-18, 250 × 4.6 mm (size), 5 µm (particle size), Thermo Fisher Scientific, Waltham, MA, USA) [24] with a mobile phase consisting of 3 mM malonic acid and 5% methanol (*v*/*v*) (pH 5.95 adjusted by tetrabutylammonium hydroxide). Arsenicals were eluted isocratically with a flow rate of 1 mL min^−1^ at 25 °C. Quality controls included the analysis of reagent blanks, spiking with the arsenic species of interest, and duplicate readings, as necessary. AsB in the samples was quantified by Clarity software (version 8.4.0.47, DataApex Ltd., Prague, Czech Republic) according to AsB standard solutions.

All samples were analyzed by these two columns to achieve an accurate analysis of AsB and all other indicated arsenic species.

### 2.8. Statistical Analysis

For mycelium growth assays, *N* = 3 was adopted. For total arsenic in mycelium and in fruiting body, *N* = 4 was adopted. Student t-test was used to calculate *p* value (Sigma Plot 10.0, Inpixon, Palo Alto, CA, USA). *p* value < 0.05 deemed as significant. For arsenic speciation studies, three replicates were collected at each treatment conditions. One compost sample was taken from each batch as arsenic is evenly distributed in the compost. Statistical analysis was used to obtain mean values and standard errors (Sigma Plot 10.0).

## 3. Results

*A. bisporus* mycelium has a relatively higher tolerance to inorganic arsenic compared with *S. cerevisiae*

**Figure 1:**
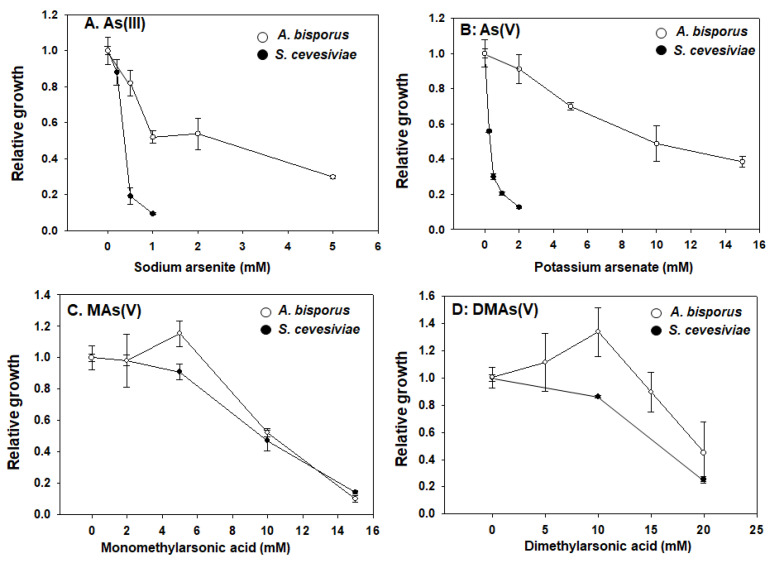
**Arsenic toxicity in *A. bisporus* mycelium and in *S. cerevisiae*.** Mycelium was purified from the commercial growing kit in malt extract agar containing chloramphenicol (50 mg/L). The purified mycelium was then aseptically grown in a liquid malt extract medium (3 mL) supplied with chloramphenicol (at final concentration of 50 mg/L) and various arsenic compounds at indicated concentrations (**A**–**D**). After growing for two weeks at room temperature, the mycelium was sonicated and OD_600_ was monitored to reflect their growth. Yeast was also tested under similar arsenic treatment conditions and used for comparison.

We tested the toxicity of four arsenic compounds in pure cultured mushroom mycelium, using *S. cerevisiae* (budding yeast) as a non-filamented fungal control. Yeast is a well adopted model organism and has well known mechanisms for arsenic toxicity and detoxification [25]. As showed in Figure 1, our results showed that different arsenic compounds have very different toxicities. When compared with yeast, mushrooms have an elevated tolerance for both inorganic species As(III) and As(V) (Figure 1A,B), while the tolerance for MAs(V) and DMAs(V) are similar and comparable to yeast (Figure 1C,D). As most of organisms, yeast showed much higher sensitivity to inorganic arsenicals.

Quantification of mycelium biomass was achieved by ultrasonic homogenizing of clustered mycelium. We found this approach to be quite replicable and efficient as it can accurately separate entangled mycelium to produce an even homogenizer to be measured.

It is challenging to determine arsenic toxicity in fruiting stage, as fruiting can be affected by many other factors and the quantification of fruiting body is not deemed as accurate. There are studies preliminary tested arsenic toxicity and accumulation in a range of 0.1–0.8 mM and showed no visible toxicity [26]. Here, we did not study arsenic toxicity in fruiting stage.

Various arsenic species are accumulated differently in two *A. bisporus* developmental stages.

**Figure 2:**
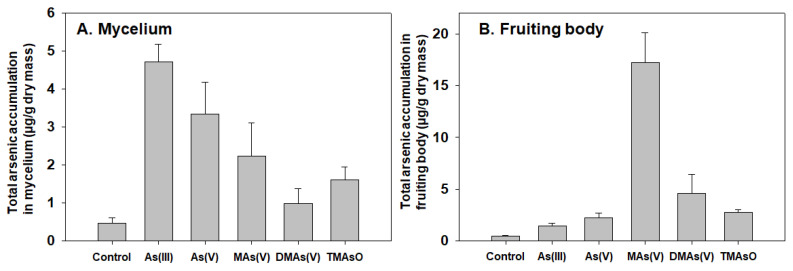
**Total arsenic accumulation in *A. bisporus* mycelium and fruiting body.** (**A**) Various arsenic compounds (indicated) were added to the aseptically cultured mycelium at final concentrations of 100 µM (in malt extract medium). After two-hour incubation, the mycelium was collected and filtered, and the total arsenic accumulated in the mycelium was quantified in each sample by ICP-MS. (**B**) To determine arsenic accumulation in fruiting bodies, various indicated arsenic compounds were added to the compost at 5 ppm (final concentration) at the beginning of culture to allow for fruiting. After 3–4 weeks, the fruiting bodies were collected, dried and weighed. Total arsenic in fruiting bodies was quantified by ICP-MS (*N* = 4 for mycelium and fruiting body samples).

We tested the arsenic accumulation of five arsenic compounds in pure cultured mycelium and in the fruiting body. Our results showed that these arsenic compounds were accumulated differently in these two stages. Inorganic As(III) and As(V) are accumulated more in mycelium when compared to the methylated species. Surprisingly, in the fruiting body, methylated species are more effectively accumulated than inorganic species. In particular, MAs(V) shows to be the most effectively accumulated species (Figure 2). All arsenic compounds were added at same final concentrations to both cultured mycelium and compost (100 μM and 5 ppm, respectively) for accurate comparison.

These results showed that environmental inorganic arsenic can be readily taken up by mycelium. Under treatment by methylated arsenic, those substrates were detected in fruiting body, suggesting those mono- and di-methylated species can be transported to and accumulated in fruiting body.

Arsenic treatment of *A. bisporus* leads to formation of mono-, di-, tri-methylated arsenic species and arsenobetaine

**Figure 3:**
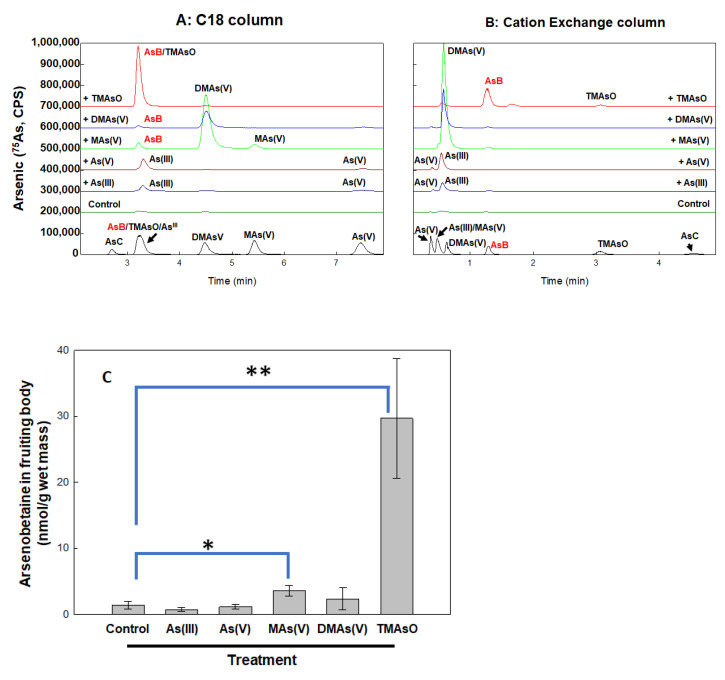
**Metabolism of various arsenic compounds in *A. biosporus*.** Various arsenic substrates were added to compost and allow for fruiting. In the fruiting body, arsenic species were determined by HPLC-ICP-MS using two different types of HPLC: C18 column (**A**) and cation exchange column (**B**) One representative data out of the three replicates is shown. AsB content under various treatment is quantified and compared (**C**) Four replicates were collected for each sample (*N* = 4). The values of AsB were calculated by the peak area (* *p* < 0.05 and ** *p* < 0.01).

The arsenic metabolites were determined in both the culture matrix (compost) containing mycelium and the fruiting body, following treatment by inorganic and methylated arsenic compounds. Each of them was supplemented to the culture matrix at 5 ppm final concentration and allowed for fruiting. Arsenic species were analyzed by HPLC-ICP-MS. Arsenic species and AsB quantification was achieved using two HPLC methods. As showed in Figure 3, the C18 column can separate all methylated species very well but the peaks of As(III), TMAsO and AsB are very close (Figure 3A). By using an ion exchange column, As(III) and AsB can be clearly separated, and TMAsO is also detectable (Figure 3B).

Our results showed that all five arsenic compounds, As(III), As(V), MAs(V), DMAs(V) and TMAsO, were metabolized differently (Figure 3A,B). As(III) was slightly oxidized to As(V), while As(V) was largely reduced to As(III).

MAs(V) treatment led to the formation of DMAs(V) as a dominant product with a trace amount of AsB, suggesting that MAs(V), which is the most efficiently accumulated species in fruiting body (Figure 2B), is an active precursor to be further methylated and modified.

In contrast, DMAs(V) was hardly metabolized, suggesting that DMAs(V) is an end product. MAs(V) appears to be an optimal precursor for further methylation and modification to from di-methylated and more complex products including AsB.

Strikingly, among the tested arsenicals, the tri-methylated TMAsO proved to be the most effective precursor for AsB formation (Figure 3C). In the fruiting body under TMAsO treatment, AsB is the major product detected. To our knowledge, this is the first reported observation of AsB biosynthesis from TMAsO in mushrooms.

Arsenobetaine is synthesized in the reproductive stage and is not affected by the addition of antibiotics in compost

**Figure 4:**
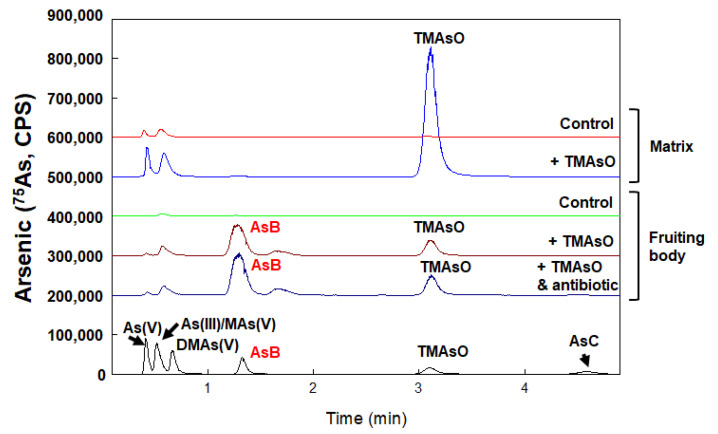
**AsB is synthesized in the reproductive but not vegetative stage and antibiotic treatment does not affect AsB synthesis.** Arsenic species in mycelium (culture matrix) and fruiting body were determined by HPLC-ICP-MS and compared in controlled culture and TMAsO treated culture. Results suggested that AsB is only synthesized in the fruiting body but not in mycelium in the culture compost when treated with TMAsO. Arsenic speciation in the fruiting body treated with TMAsO were also compared with the chloramphenicol treated culture (at final concentration of 50 mg/Kg in compost). Results showed that AsB was detected in both conditions with comparable levels. One representative data out of the three replicates is shown.

We designed studies to explore in which stage of white button mushrooms AsB is synthesized (Figure 4). The arsenic speciation in the fruiting body and mycelium were compared in both a controlled culture (without arsenic treatment) and TMAsO treated samples. We noticed that in TMAsO treated fruiting body samples, AsB was not detected in the mycelium-containing culture matrix and TMAsO remained to be the dominant species (Figure 4, +TMAsO, Matrix). However, in the fruiting body, both TMAsO and AsB exist, with AsB as the major metabolite. This result suggested that TMAsO could be transported to fruiting body, then metabolized to form AsB. Therefore, AsB formation could be strictly phase-dependent and only formed in the reproductive stage.

To test whether symbiotic microbes play a role in arsenic metabolism, mushrooms were cultured with chloramphenicol. The concentration of chloramphenicol we used for the malt extract medium was the same concentration used for mycelium purification. Although it is not a completely aseptical condition, the addition of antibiotics limited microbial growth. As depicted in Figure 4, the formation of AsB with the addition of antibiotics show the same pattern. We tentatively conclude that AsB formation is catalyzed by mushroom enzymes rather than by environmental microbes.

## 4. Discussion

The mushroom represents unique species of fungi that can form fruiting bodies. They are well-known for their nutrients, medical application, as well as production of fungal toxicants [27,28]. They are generally recognized as a hyper accumulator of heavy metals [29]. One unique property of mushrooms in regard to arsenic detoxification is that they can produce non-toxic AsB. This property renders mushroom an interesting organism to investigate arsenic metabolism, with a promising lead to new strategies for management of arsenic contamination. However, the mechanisms for AsB biosynthesis remains unknown. The precursors, the reactions, and the genes/enzymes that are involved in AsB formation have not been characterized well, especially in mushrooms.

Our studies aim to explore the conditions and pathways for AsB synthesis with a long-term goal to elucidate the molecular mechanisms. The white button mushroom, *A. bisporus,* was chosen as a culturable mushroom model. Belonging to the *Basidiomycota* class, *A. bisporus* is the most commercialized dietary mushroom. The genome map of *A. bisporus* was drafted in 2012 with a genome size of 30 Mbp. There are approximately 10,000 genes in the genome, yet most of the gene functions are not known.

Unlike the published report [30], no AsB was detected under As(V) treatment. This could be caused by variation of strains and culture conditions. While As(III), As(V), and DMAs(V) were hardly methylated, MAs(V) was largely converted to DMAs(V), suggesting that arsenic methylation is an active metabolism pathway, with DMAs(V) representing a stable end product of methylation. We speculate that mushrooms contain a methyltransferase that can methylate MAs(V) into DMAs(V). We also found that MAs(V) serves as the most effectively accumulated species in fruiting body (Figure 2B).

Most strikingly, we found that when trimethylated arsenic species TMAsO was applied, the major arsenic product was AsB in the fruiting body. This is the first time that the TMAsO is found to be the optimal precursor for AsB formation. These results showed that arsenic methylation is not only a dominant pathway to produce methylated arsenic metabolites, but it can also generate precursor(s) for AsB formation. Therefore, we propose that these two pathways are connected, as illustrated in the graphic abstract.

As the only non-toxic arsenic species, the biosynthesis of AsB has attracted huge interest since it was first detected in marine fish [31]. AsB is a two-carbon organic acid containing arsenic with three methyl functional groups. Its nitrogen-containing homologue glycine betaine is commonly used as an osmolyte in nature [32]. While some preliminary studies in marine fish were conducted beginning in the 1990s, there is still no scientific consensus about the precise biosynthetic pathways of arsenobetaine. In fish, it has been hypothesized that AsB is degraded from fish food algae containing arsenosugars [16]. In *Bacillus subtilis*, which utilizes AsB to protect themselves from high osmolarity and extremes in temperatures, AsB can be synthesized from AsC by two sequential steps that are catalyzed by two enzymes, GbsB (choline dehydrogenase) and GbsA (glycine betaine aldehyde dehydrogenase) encoded in gbs (glycine betaine synthesis) operon [33]. Our results, for the first time, clearly show that AsB in mushrooms is synthesized from TMAsO, not from arsenosugar nor from arsenocholine. TMAsO was detected in many mushroom species [17,34]. The efficient conversion of TMAsO to AsB suggests that a unique pathway for AsB synthesis should exist in mushrooms, one that should add two-carbon substrates to TMAsO, forming a new As-C bond. Such mechanism requires further investigation.

Our results also support that AsB synthesis occurs in the fruiting body, as it is not detected in culture matrix (Figure 4). We predict that there is(are) development-dependent enzyme(s) expressed in the fruiting phase that convert(s) TMAsO to AsB.

*A. bisporus* is deemed symbiotic, requiring collaborating environmental microbes for fruiting [35]. There have been debates over the roles of the communizing microbes in mushroom arsenic biotransformation [15]. Because fruiting strictly requires community microbes and/or their metabolites, the completely aseptical conditions during the mushroom growing process are challenging to achieve. Our results showed that the reduction of microbial activities in compost does not affect AsB formation (Figure 4). This suggests that AsB biosynthesis is likely catalyzed by enzyme(s) that is(are) expressed in the mushroom itself, rather than in symbiotic microbes. We also tried to culture mushrooms using autoclaved casing, but no fruiting was achieved (data not shown).

At present, high-performance liquid chromatography-coupled inductively coupled mass spectrometer (HPLC-ICP-MS) is the most accurate technique for arsenic speciation and quantification [34]. This technique utilizes HPLC to separate arsenic compounds and uses ICP-MS to detect ^75^As peaks. As arsenic metabolites in mushrooms include multiple compounds of similar properties, each separation approach has some limitations and cannot satisfy the full range analysis of arsenic species. Therefore, we adopted two HPLC separation columns—an ion exchange column and a hydrophobic column, which allowed us to achieve desirable analysis results [20,23]. The reduced forms of methylated arsenicals were not detected. The detection of stable AsB and various methylated species shows high resolution.

Although previous studies examined the arsenic accumulation in fruiting body under a range of inorganic arsenic treatment [26], we show here for the first time that the stage-dependent differential accumulation patterns of inorganic and methylated arsenic species, which suggest that in mushrooms, the inorganic arsenic, which is well tolerated, would be taken up by the mycelium readily and the methylated species are then transported to fruiting body. This mechanism will allow the environmental inorganic arsenic to be accumulated and metabolized by mushrooms efficiently.

Elucidation of the molecular mechanisms of AsB formation still awaits future studies, but our results provide a new foundation to identify those genes. Exploration of AsB synthesis pathway can be assisted by using the current transcriptomic, proteomics, and metabolomics technique. The elucidation of the AsB biosynthesis mechanisms will provide a promising solution to construct engineered microbes for arsenic bioremediation in the future.

## 5. Conclusions

We designed experiments to determine the metabolism of various arsenic compounds in lab cultured white button mushroom *A. bisporus*, with a goal to find possible arsenobetaine biosynthesis pathways. Our results, for the first time, showed that tri-methylated arsenic TMAsO can be converted efficiently to AsB and this result suggested a route for arsenobetaine biosynthesis which involves two connected pathways: arsenic methylation and arsenobetaine formation.

We also found white button mushroom has higher tolerance to inorganic arsenic compared to fungal yeast, and the accumulation of methylated arsenic and inorganic arsenic species showed opposite patterns in the vegetative phase and reproductive phase.

## Abbreviations

SAM	S-adenosyl methionine
AsB	arsenobetaine
AsC	arsenocholine
HPLC-ICP-MS	high performance liquid chromatography coupled inductively coupled mass spectrometer (ICP-MS)
MAs(V)	methylarsenate
DMAs(V)	dimethylarsonate
As(III)	arsenite
As(V)	arsenate
TMAsO	Trimethylarsine oxide
